# Correlation between immune-related adverse events and the efficacy of PD-1/PD-L1 inhibitors in the treatment of non-small cell lung cancer: systematic review and meta-analysis

**DOI:** 10.1007/s00280-021-04375-2

**Published:** 2021-11-25

**Authors:** Qian Zhang, Wei Wang, Qi Yuan, Li Li, Yu-Chao Wang, Chuan-Zhen Chi, Chun-Hua Xu

**Affiliations:** 1 Department of Respiratory Medicine, Nanjing Chest Hospital, 215 Guangzhou Road, Nanjing, 210029 China; 2 Affiliated Nanjing Brain Hospital, Nanjing Medical University, Nanjing, 210029 Jiangsu China; 3 Clinical Center of Nanjing Respiratory Diseases and Imaging, Nanjing, 210029 Jiangsu China

**Keywords:** Non-small cell lung cancer, PD-1, PD-L1, Inhibitor, Immune-related adverse event, Efficacy

## Abstract

**Objective:**

Anti-programmed cell death-1 and programmed cell death ligand-1 (PD-1/PD-L1) inhibitors have been proved to have a significant clinical efficacy in the treatment of non-small cell lung cancer (NSCLC). Many studies have demonstrated that immune-related adverse events (irAEs) are significantly correlated with clinical efficacy, but the results are not consistent. This meta-analysis aimed to evaluate the associations between irAEs and efficacy.

**Methods:**

Comprehensive searches were conducted on PubMed and EMBASE database. The HR and 95% CI were used to assess the associations between immune-related adverse events and efficacy of overall survival and progression-free survival. Subgroup analyses were performed based on irAEs type and grade of irAEs. Heterogeneity and publication bias were also assessed by *Q* test, *I*^*2*^, and funnel plot.

**Results:**

Compared with non-irAEs, the development of irAEs was significantly improved PFS and OS (PFS: HR = 0.55, 95% CI = 0.51–0.60, *p* < 0.001; OS: HR = 0.74, 95% CI = 0.68–0.81, *p* < 0.001). In the subgroup analyses, the occurrence of endocrine irAEs, gastrointestinal irAEs, skin lesions and low-grade irAEs was also significantly correlated with the efficacy. Additionally, the association between severe-grade irAEs and survival benefits on PFS was significant, but not on OS.

**Conclusions:**

The results indicated that the occurrence of irAEs was significantly associated with a better efficacy in the treatment of NSCLC, especially endocrine, gastrointestinal, skin and low-grade irAEs.

## Introduction

PD-1/PD-L1 inhibitors were established as an important component in the field of immunotherapy for non–small cell lung cancer (NSCLC). Many retrospective studies have demonstrated that immune checkpoint inhibitors (ICIs) dramatically improved long-term survival in treated patients with advanced NSCLC [1–3]. Compared to anticancer therapies, the ICIs may cause immune-related adverse events (irAEs) because of nonspecific immune activation [4]. Regarding the mechanisms of ICIs, while immune cells attack tumor cells, it also promotes the immune system to attack normal tissues and organs. IrAEs can involve almost every organ of the body, but the skin, gastrointestinal tract, pulmonary and endocrine are the most common organs [5]. Despite the good clinical efficacy, but in the clinical treatment, the development of irAEs greatly limits the application of ICIs in many cancer patients.

Several studies reported the occurrence of irAEs could improve survival outcomes with advanced NSCLC [6, 7], but in the other reports, the correlation has not been investigated [8]. Therefore, it is still controversial whether the presence of irAEs is the predictive factors of the ICI response in advanced NSCLC. A systematic review has supported the relationship between irAEs occurrence and the curative effect of ICIs in all solid malignancies [9]. To explore the associations of the development of irAEs and the curative effect in advanced NSCLC, we conducted a meta-analysis of published data. The predictive effects of different irAEs types, irAEs grades and the impact on outcome were analyzed.

### Materials and methods

## Literature source and search strategy

Published studies were searched on PubMed and EMBASE databases to investigate the associations between irAEs occurrence and ICIs efficacy in patients with advanced NSCLC (Database inception to December 1, 2020). The keywords of this study were ‘‘irAEs or immune-related adverse events” and ‘‘lung cancer”. Language is limited to English. In addition, the retrieved literatures were also searched manually. Inclusion criteria for this meta-analysis have to meet the following: (1) The subjects were diagnosed with lung cancer and received at least one PD-1/PD-L1 inhibitors; (2) Studies that reported the relationship between irAEs and curative effect in NSCLC; (3) Studies included hazard ratios (HRs) of OS and PFS, as well as available survival data of HRs and 95% confidence intervals (CIs) or *p* values; (4) Prospective or retrospective cohort studies.

### Data extraction

The data were extracted by two investigators independently. The third reviewer checks the date again if the data is inconsistent. For each included study, we extracted the year of publication, the first author’s name, PD-1 or PD-L1 antibodies, trial design, statistical model, type of irAEs, grade of irAEs, HRs and 95% CIs of OS and PFS in patients with irAEs, HRs and 95% CIs of OS and PFS in patients without irAEs. HRs and 95% CIs of OS and PFS for global irAEs, HRs and 95% CIs of OS and PFS for each organ irAEs, HRs and 95% CIs of OS and PFS for each grade irAEs. If the study included both univariate and multivariate HRs, the multivariate HRs was selected.

### Statistical analysis

All the data statistical analyses and plotting were implemented with 15.0 Stata software (USA). The strength of the relationship between irAEs occurrence and the efficacy of ICIs was calculated by pooled HRs and 95% confident interval (CI). The impact of research size on the results was evaluated by Weight. The pooled HRs of irAEs versus non-irAEs and 95% CIs were adopted to summarize the survival results (*p* < 0.05 was considered significant). The *χ*^2^ test and *I*^2^ statistic were used to estimate the heterogeneity between the studies. If *p* < 0.05 of the *χ*^2^ test or *I*^2^ > 50% indicated that there is significant heterogeneity, the meta-analysis use a random-effects model [10]. Otherwise, the fixed-effects model will be used [11]. Publication bias was tested by the Funnel plot and Egger’s linear regression test [12]. All statistical analyses were considered representative of statistical significance for a two-sided *p* < 0.05. Subgroup analysis was also conducted by a type of irAEs and grade of irAEs.

### Results

### Characteristics of studies

**Fig. 1:**
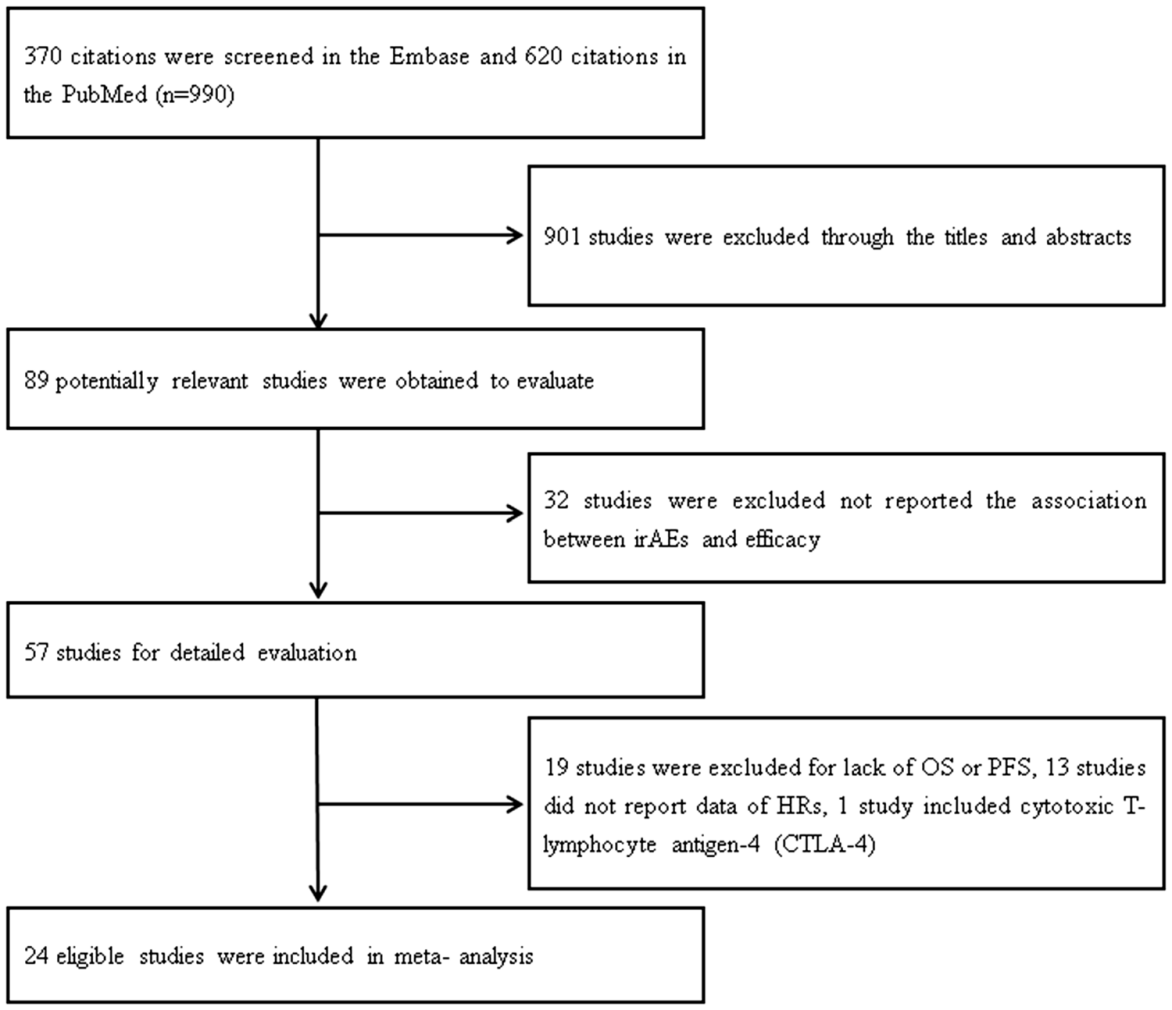
Flowchart and the detailed process of eligible studies

We searched a total of 990 studies, and after sifting through the titles and abstracts, 89 potentially eligible studies might be eligible. 32 studies that did not report the relationship between irAEs and efficacy were excluded. 19 studies were excluded due to lack of OS or PFS, 13 studies did not report HRs data, 1 study was excluded because it included CTLA-4. Finally, a total of 24 studies were included in this meta-analysis [6, 13–35]. Figure 1 shows the specific retrieval process. Table 1 summarizes the detailed characteristics of the eligible studies. Among these studies, 24 studies reported total irAEs, and 12 studies reported irAEs for individual organs. 23 studies reported the grade of irAEs. 20 studies adopted the drugs of anti-PD-1/PD-L1 inhibitors. 20 studies reported HRs of OS and 20 studies reported HRs of PFS. Prospective cohort design was used in 2 studies and retrospective cohort design was used in 22 studies. 15 studies adopted multivariate models, and univariate models were used in 9 studies.

**Table 1:** Main characteristics of the included articles

Study	PD-1or PD-L1	irAE grade	irAE type	HR for PFS (95%CI)	HR for OS (95%CI)	Model	Design
Kim 2017	Nivolumab Pembrolizumab	1–2	Thyroid dysfunction	0.38 (0.17–0.85)	0.11 (0.01–0.92)	M	RC
Osorio [13]	Pembrolizumab	1–3	Thyroid dysfunction	0.58 (0.27–1.21)	0.29 (0.09–0.94)	U	PC
Haratani 2017	Nivolumab	1–4	Any irAE	0.542 (0.295–0.971)	0.285 (0.102–0.675)	M	RC
		1–4	Skin	0.476 (0.232–0.912)	0.209 (0.049–0.618)		
		1–4	Endocrine	0.237 (0.037–0.842)	0.504 (0.027–2.629)		
Grangeon 2018	Anti-PD-L1 or anti-PD-1	Any grade	Any irAE	0.42 (0.32–0.57)	0.29 (0.18–0.46)	U	RC
		Any grade	Pneumonitis	1.19 (0.52–2.70)	1.42 (0.45–4.54)		
		Any grade	Colitis	0.73 (0.35–1.50)	0.24 (0.03–1.73)		
		Any grade	Hepatitis	0.97 (0.45–2.08)	0.97 (0.30–3.08)		
		Any grade	Thyroiditis	0.58 (0.39–0.85)	0.46 (0.25–0.86)		
Toi [7]	Nivolumab or pembrolizumab	1–4	Any irAE	0.45 (0.30–0.68)	0.42 (0.24–0.71)	U	RC
Sato [14]	Nivolumab	1–4	Any irAE	0.28 (0.04–1.46)		U	RC
Ricciuti [22]	Nivolumab	1–4	Any irAE	0.48 (0.34–0.67)	0.38 (0.26–0.56)	M	RC
		1–4	Lung	0.56 (0.33–0.96)	0.46 (0.24–0.89)		
		1–4	Gastrointestinal	0.52 (0.3–0.9)	0.5 (0.26–0.98)		
		1–4	Endocrine	0.59 (0.4–0.89)	0.45 (0.28–0.72)		
		1–4	Skin	0.57 (0.35–0.95)	0.8 (0.46–1.39)		
		1–4	Hepatobiliary	0.72 (0.41–1.42)	0.94 (0.53–1.66)		
Ksienski [20]	Nivolumab and pembrolizumab	1–2	Any irAE		0.85 (0.50–1.42)	M	
		> 3	Any irAE		2.29 (1.05–4.98)		
	Nivolumab	1–2	Any irAE		0.74 (0.41–1.31)		
		≥ 3	Any irAE		2.53 (1.15–5.57)		
Lesueur [19]	Nivolumab	1–4	Any irAE	0.660 (0.433–1.099)	0.64 (0.377–1.087)	M	RC
Lisberg [16]	Pembrolizumab	1–4	Any irAE	0.62 (0.40–0.96)	0.72 (0.49–1.05)	M	RC
Fujimoto [17]	Nivolumab	≥ 3	Any irAE	0.76 (0.55–1.01)		M	
		1–4	Pneumonitis	0.71 (0.52–0.97)		M	RC
Cortellini [24]	Anti-PD-1	1–4	Any irAE	0.59 (0.47–0.76)	0.55 (0.41–0.72)	M	RC
		3–4	Any irAE	0.75 (0.51–1.11)	0.76 (0.48–1.21)	M	
		1–4	Endocrine	0.63 (0.45–0.89)	0.55 (0.37–0.83)	M	
		1–4	Skin	0.46 (0.31–0.69)	0.43 (0.27–0.70)	M	
		1–4	Gastrointestinal	0.68 (0.47–1.01)	0.61 (0.38–0.98)	OS: M PFS: U	
		1–4	Pneumonitis	1.20 (0.76–1.92)	1.32 (0.79–2.19)	U	
		1–4	Hepatobiliary	1.47 (0.72–1.96)	1.09 (0.48–2.45)	U	
Ahn, [25]	Nivolumab or pembrolizumab	1–4	Any irAE	0.434 (0.256–0.735)	0.484 (0.255–0.919)	M	RC
		1–2	Skin	0.643 (0.350–1.180)	0.42 (0.162–1.087)		
		1–4	Endocrine	0.368 (0.132–1.028)	0.255 (0.051–1.288)		
		1–4	Pneumonitis	1.686 (0.618–4.579)	4.177 (1.420–11.942)		
Berner [26]	Anti-PD-1	NA	Skin	0.22 (0.09–0.49)	0.29 (0.12–0.71)	U	PC
Bjørnhart 2019	ICI	3–4	Any irAE	0.71 (0.39–1.27)	0.47 (0.21–1.05)	U	RC
Imai 2019	Embrolizumab	1–4	Any irAE	0.70 (0.35–1.37)	0.78 (0.28–1.37)	U	RC
Baldini [28]	Nivolumab	1–4	Any irAE		1.44 (1.22–1.71)	M	RC
Ksienski [29]	Pembrolizumab or nivolumab	1–5	Any irAE		1.37 (0.91–2.08)	M	RC
Sugano [30]	Nivolumab, pembrolizumab or atezolizumab	1–4	ILD	0.39 (0.19–0.77)		M	RC
Naqash [31]	Nivolumab	Any	Any irAE	0.69 (0.55–0.87)	0.62 (0.55–1.03)	M	RC
		Any	Thyroid dysfunction	0.98 (0.67–1.42)	0.79 (0.53–1.19)	U	
		Any	Pneumonitis	1.36 (0.91–2.02)	1.35 (0.89–2.02)	U	
		Any	Hepatitis	0.75 (0.45–1.31)	1.18 (0.63–1.97)	U	
		Any	Colitis/diarrhea	0.65 (0.35–1.21)	0.65 (0.35–1.21)	U	
		Any	Musculoskeletal	0.31 (0.04–1.87)	0.37 (0.11–1.17)	U	
		Any	Skin	0.55 (0.34–0.87)	0.67 (0.41–1.07)	OS: U PFS: M	
Yamaguchi [32]	Pembrolizumab or nivolumab	Any grade	Any irAE	0.73 (0.48–1.09)	0.83 (0.51–1.32)	U	RC
Cortellini [33]	Pembrolizumab	Any	Any irAE	0.49 (0.39–0.61)	0.41 (0.31–0.53)	M	RC
		3–4	Any irAE	0.78 (0.57–1.05)	0.70 (0.48–1.03)	U	
		Any	Cutaneous	0.72 (0.51–1.01)	0.48 (0.30–0.78)	M	
		Any	Endocrine	0.40 (0.27–0.59)	0.30 (0.17–0.52)	M	
		Any	Gastrointestinal	0.58 (0.39–0.86)	0.67 (0.42–1.07)	OS: U PFS: M	
		Any	Hepatic	1.31 (0.83–2.06)	0.82 (0.43–1.54)	U	
		Any	Pulmonary	0.65 (0.39–1.09)	0.59 (0.30–1.14)	U	
		Any	Rheumatlogic	0.50 (0.29–0.87)	0.47 (0.23–0.96)	M	
		Any	Neuro-muscular	0.50 (0.18–1.34)	0.52 (0.16–1.62)	U	
Noguchi [34]	Pembrolizumab	Any grade	Any irAE	0.33 (0.17–0.65)		M	RC
Kubo [35]	Nivolumab/pembrolizumab	Any grade	Any irAE		1.59 (0.93–2.71)	U	
		≥ 2	Any irAE		1.18 (0.70–1.99)	U	RC

*OS* overall survival, PFSprogression-free survival, *M* multivariate, *U* univariate, RCretrospective cohort, *PC* prospective cohort

### Progression-free survival

**Fig. 2:**
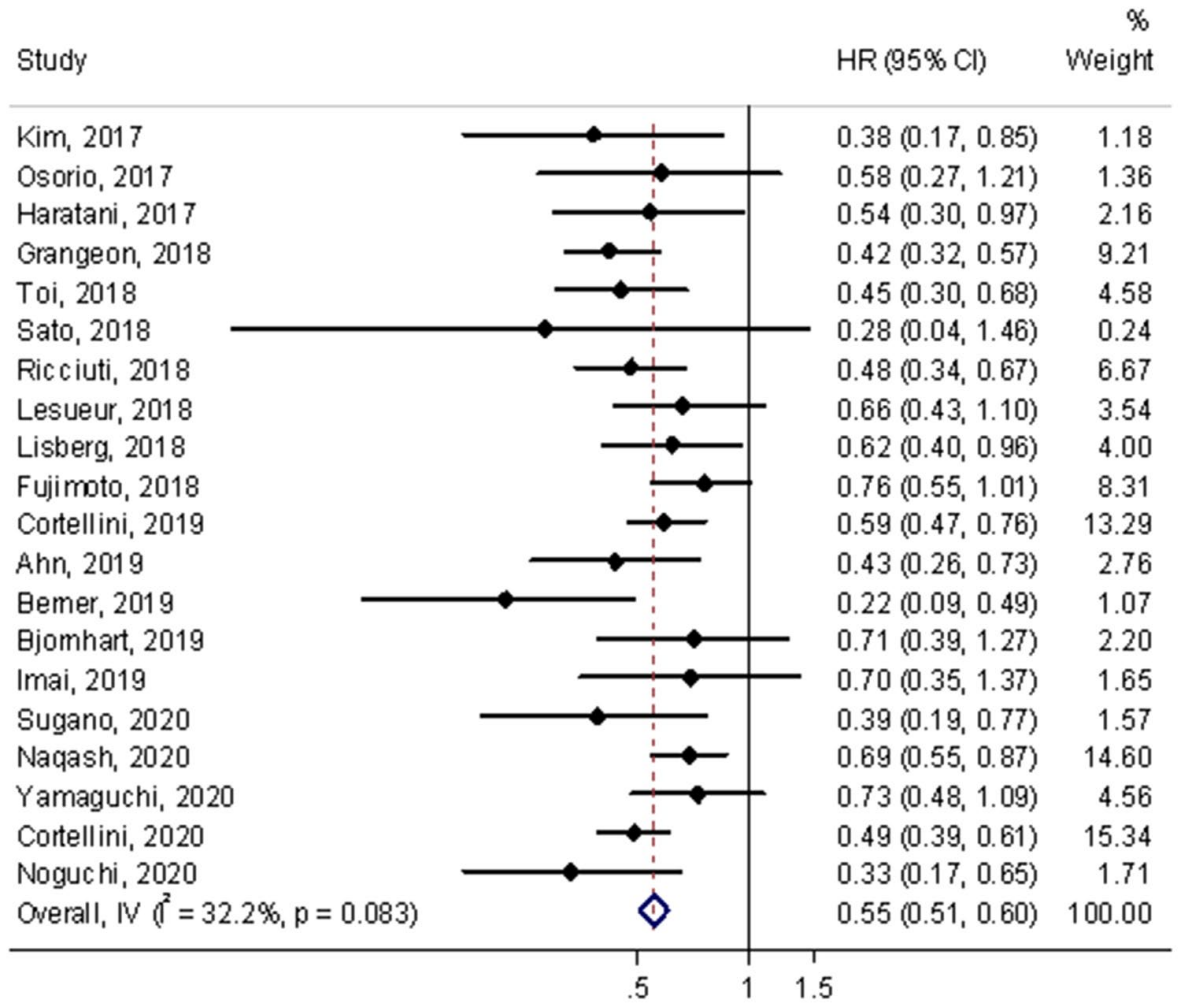
Forest plot of immune-related adverse event development associated with PFS. The diamond represents the summary HR and 95% CI

A total of 20 studies assessed HRs of PFS in the meta-analysis [6, 13–19, 21–27, 30–34]. The results showed that PFS was significantly improved for the occurrence of irAEs compared with non-irAEs (HR = 0.55, 95% CI = 0.51–0.60, *p* < 0.001; shown in Fig. 2). No heterogeneity was observed among studies for the occurrence of irAEs and PFS in the pooled analysis (*I*^2^ = 32.2%, *p* = 0.083).

Subgroup analysis was performed according to the irAEs types, a significant association was observed between the occurrence of endocrine (HR = 0.59, 95% CI = 0.50–0.69), gastrointestinal (HR = 0.62, 95% CI = 0.50–0.77, *p* < 0.001), skin lesions (HR = 0.56, 95% CI = 0.46–0.68, *p* < 0.001) and improved PFS in patients treated with ICIs. Nevertheless, significant associations were not found in the occurrences of pulmonary irAEs (HR = 0.85, 95% CI = 0.71–1.01, *p* = 0.058) and hepatobiliary irAEs (HR = 1.06, 95% CI = 0.83–1.35, *p* = 0.654) with PFS. No significant heterogeneity was observed in endocrine, gastrointestinal, skin lesions and hepatobiliary irAEs, but was observed in pulmonary irAEs (*I*^2^ = 63.8%, *p* = 0.007). According to the grades of irAEs, patients with severe-grade had higher response rates. Low-grade irAEs were also significantly associated with a good PFS (Table 2). No significant heterogeneity was observed both in severe-grade and low-grade.

**Table 2:** Subgroup analyses of the association between immune-related adverse events and PFS

	HR(95%CI)	*p*	*P*_heterogeneity_	*I*^2^ (%)
IrAEs type				
Endocrine	0.59 (0.50–0.69)	< 0.001	0.076	43.8%
Gastrointestinal	0.62 (0.50–0.77)	< 0.001	0.918	0.0%
Hepatobiliary	1.06 (0.83–1.35)	0.654	0.229	28.9%
Pulmonary	0.85 (0.71–1.01)	0.058	0.007	63.8%
Skin	0.56 (0.46–0.68)	< 0.001	0.163	36.6%
IrAEs grade				
Low-grade(1–2)	0.53 (0.33–0.86)	0.01	0.311	2.6%
Severe-grade(≥ 3)	0.76 (0.64–0.91)	0.003	0.994	0.0%

### Overall survival

**Fig. 3:**
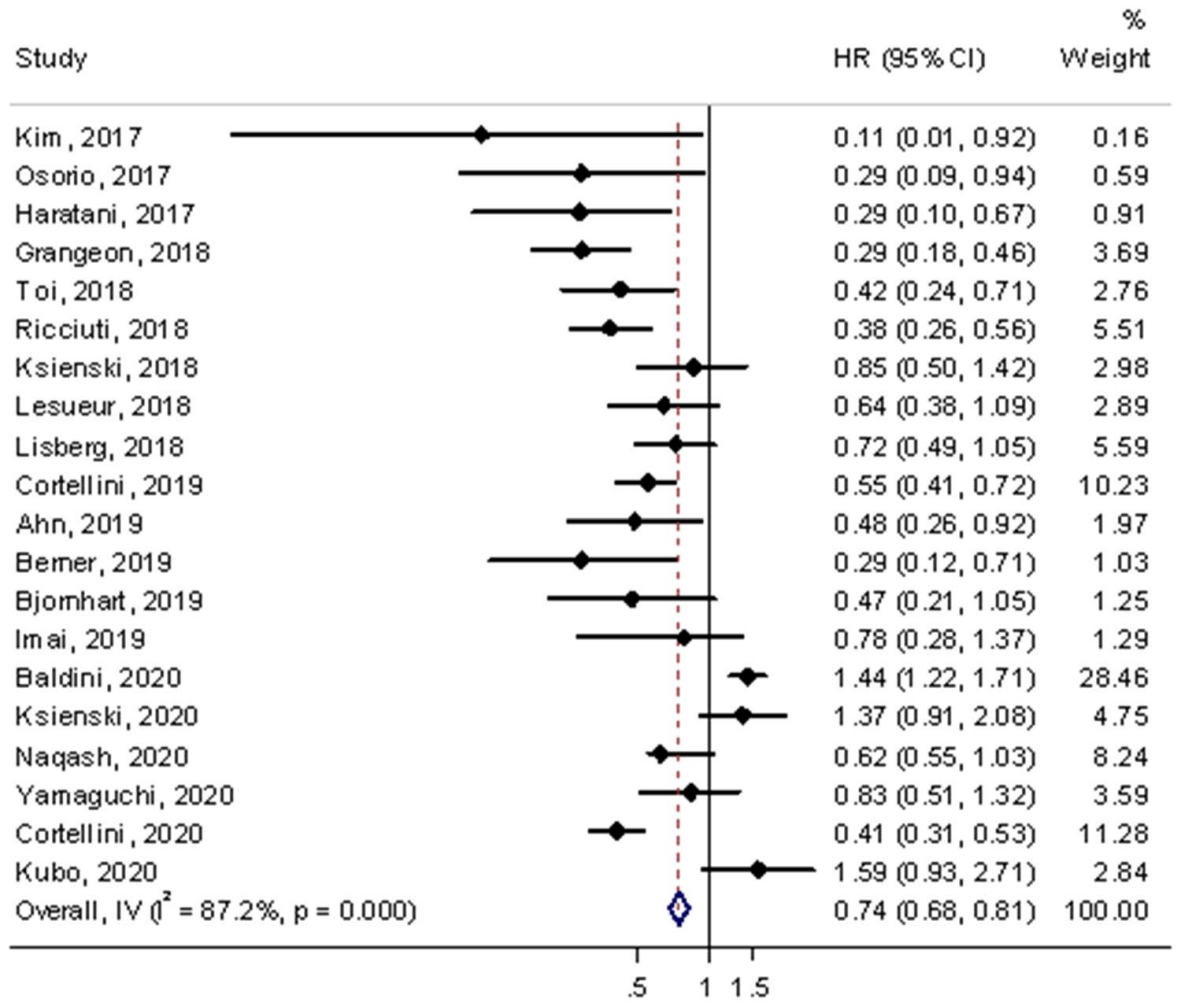
Forest plot of immune-related adverse event development associated with OS. The diamond represents the summary HR and 95% CI

A total of 20 studies assessed HRs of OS in the meta-analysis [6, 13, 15, 16, 18–29, 31–33, 35]. The results showed that irAEs was significantly associated with favorable OS compared with non-irAEs which is similar to PFS (HR = 0.74, 95% CI = 0.68–0.81, *p* < 0.001; shown in Fig. 3). However, significant heterogeneity was accompanied in the pooled analysis (*I*^2^ = 87.2%, *p* < 0.001).

In the subgroup analysis of irAEs types, similar with PFS, the occurrence of endocrine, gastrointestinal and skin were significantly associated with improved OS (endocrine: HR = 0.50, 95% CI = 0.41–0.62, *p* < 0.001; gastrointestinal: HR = 0.61, 95% CI = 0.47–0.79, *p* < 0.001; skin: HR = 0.53, 95% CI = 0.42–0.67, *p* < 0.001). However, significant associations were not detected in pulmonary and hepatobiliary irAEs with a favorable OS. Significant heterogeneity was observed in pulmonary, but not in endocrine, gastrointestinal and skin. Stratified analysis according to the grades of irAEs indicated that severe-grade was not significantly associated with favorable OS, but a favorable OS was observed in low-grade irAEs (Table 3). Significant heterogeneity was not observed in low-grade but was detected in severe-grade.

**Table 3:** Subgroup analyses of the association between immune-related adverse events and OS

	HR(95%CI)	*p*	*P*_heterogeneity_	*I*^2^ (%)
IrAEs type				
Endocrine	0.50 (0.41–0.62)	< 0.001	0.160	32.2%
Gastrointestinal	0.61 (0.47–0.79)	< 0.001	0.853	0.0%
Hepatobiliary	0.99 (0.73–1.35)	0.971	0.940	0.0%
Pulmonary	1.09 (0.85–1.40)	0.497	0.003	71.9%
Skin	0.53 (0.42–0.67)	< 0.001	0.311	16.0%
IrAEs grade				
Low-grade(1–2)	0.70 (0.49–0.99)	0.045	0.236	29.3%
Severe-grade(≥ 3)	0.93 (0.74–1.16)	0.531	0.003	71.8%

### Tests for sensitivity and publication bias

**Fig. 4:**
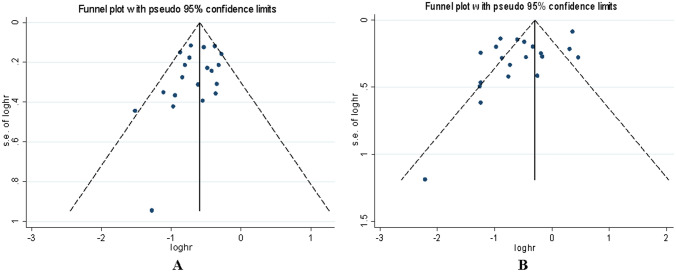
Funnel plot for the publication bias (**A**) PFS. (**B**) OS. Each point represents a separate study for the indicated association

We did not find that a single study can change the pooled results for in the sensitivity analysis, which indicated that the significant association between irAEs and PD-1/PD-L1 inhibitors efficacy was stable. In the meta-analysis, the publication bias was assessed by Begg funnel plot and Egger’s test. The Begg funnel plot did not show significant asymmetry for PFS (*p* = 0.256) (Fig. 4A). In addition, the results of Egger’s test did not show any evidence of publication bias (*p* = 0.160). Regarding OS, the shape of the Begg funnel plot did not show obvious asymmetry (*p* = 0.770) (Fig. 4B), but Egger’s test showed publication bias (*p* = 0.029), indicating that publication bias was detected for OS. Then, the trim and fill method was used to certificate the effect of publication bias on the pooled results, which further proved that the results are stable.

## Discussion

We all know that the immune system plays a very important role in the progression and treatment of cancer. PD-1/PD-L1 receptor blocker by inhibiting the escape of cancer cells from host T-cells which has become a new immunotherapy for malignant [36]. The application of immunotherapy, especially of PD-1/PD-L1 inhibitors, provides unprecedented curative effect for the treatment of NSCLC. However, in the process of activating host T cells against malignant antigen tissues, inhibition checkpoint blocking may also attacks on other tissues [37]. Consequently, with the promotion of monotherapy and combination therapy, unpredictable efficacy and inevitable irAEs are two problems which increasingly obvious. At present, whether the occurrence of irAEs is related to the treatment of ICI remains controversial. This study provides a more comprehensive and widespread analysis of the relationship between irAEs and the treatment efficacy of ICI.

In the analysis, we found that compared with patients who were without irAEs, patients who developed irAEs experienced a longer OS and PFS. In addition, the correlation was very stable, and there was no significant change in a sensitivity analysis. So far, the machine-processed between irAEs and survival benefits is not fully clear. The most promising hypotheses for this phenomenon could be the Antigen mimicry theory between tumor and healthy tissue [26]. Immune checkpoint is an important part of the molecular mechanism of maintaining peripheral immune tolerance. The release of antigens by ICI therapy is considered as one of the prime mechanisms that can trigger irAEs [38]. Thus, the development of irAEs indicates that irAEs have a strong immune response to both tumor and healthy tissues, thereby predicting a better therapeutic response. The results indicated that irAEs might be a predictive factor of durable efficacy in NSCLC.

The stratified analysis based on irAEs types. The results indicate that endocrine irAEs, skin irAEs and gastrointestinal irAEs have favorable results. However, no significant associations were found between the hepatobiliary irAEs, pulmonary irAEs and favorable results in NSCLC. Previous study have suggested that among the patients treated with immune checkpoint inhibitors, 14%–47% of the patients will have skin reactions, the severity of these reactions varies from mild to widespread, and 1%–3% of the patients will have this reaction [39]. About 4%–10% or more NSCLC patients who were treated with nivolumab have rashes and itching [40]. According to the report, Pembrolizumab leads to cutaneous reactions in about 9%–27% of patients [41]. ASO et al. [42] found that early skin reactions within 6 weeks seem to be related to the efficacy of ICI therapy which had better ORR and PFS than patients without skin reaction. Thyroid dysfunction is the most common endocrine irAEs. The mechanism of thyroid dysfunction during immunotherapy is not well understood. The investigators hypothesized that thyroid toxicity occur because of either humoral immunity or deterioration of low-level autoimmunity during anti-PD-1/PD-L1 antibody therapy [13]. Zhou et al. [9] found that the favorable results remained insignificant for endocrine and gastrointestinal irAEs might be explained by heterogeneity which is inconsistent with our study. According to hepatobiliary irAEs and pulmonary irAEs, considering that tumors of respiratory and hepatobiliary systems are the most commonly involved in anti-PD-1/PD-L1 therapy, it may increase mortality and lead to undesirable results [43].

Regarding the subgroup analyses based on irAEs grades, there was significant prognostic value on low-grade irAEs. The prognostic value was also significant on severe-grade irAEs for FPS. But no significant associations were found between the severe-irAEs and favorable OS on severe-grade irAEs. First, fewer patients are considered to have grade 3 or higher grade, it does not have sufficient capacity to determine any correlation. Second, because patients with severe irAEs may be life-threatening, glucocorticoid therapy is required to save lives which inhibit the effect of ICI and promote the growth of tumor [44]. Therefore, accurate assessment of tumor response is considered more difficult.

Our meta-analysis has some limitations that need to be improved. First of all, our study only includes published studies, and many unpublished data are not included, and we excluded several studies because they did not report HR values and other reasons. Therefore, publication bias is hard to avoid, Egger’s test indicated the existence of publication bias in the results of OS. But, the trim and fill method and Begg’s test further prove the stability. Second, there existed significant heterogeneity in the OS analysis, which might result from irAEs types and irAEs grades. To reduce the effect of heterogeneity, we analyzed each type and grade of irAEs. Third, due to limited resources, subgroup analysis was not performed according to the anti PD-1 and PD-L1 antibody types. Due to the lack of detailed analysis of tumor staging, class of ICIs, combination therapy and treatment line, which may influence the results of our study. Finally, because our study included a limited number of studies, therefore, the statistical ability is weak in the evaluation of the correlation between the irAEs development and the survival benefit of anti-PD-1/PD-L1 antibody, especially in stratified analyses.

In conclusion, our study further demonstrated that the development of irAEs with anti-PD-1/PD-L1 antibody therapy is related to better survival benefits in patients with NSCLC, especially endocrine, gastrointestinal, skin and low-grade irAEs. With the rapid development of immunotherapy, it will become very important to find the indicators to predict the efficacy. Our results suggest that irAEs may be a potential prognostic factor for efficacy. However, due to the small number of studies, some results are limited. Therefore, it is necessary to conduct further large-scale research.
